# Evaluation of the Efficacy of Doxycycline, Ciprofloxacin, Levofloxacin, and Co-trimoxazole Using *In Vitro* and *In Vivo* Models of Q Fever

**DOI:** 10.1128/AAC.00673-21

**Published:** 2021-10-18

**Authors:** K. A. Clay, M. G. Hartley, S. Armstrong, K. R. Bewley, K. Godwin, E. Rayner, J. Vipond, M. Bailey, T. P. Atkins, I. H. Norville

**Affiliations:** a Academic Department, Royal Centre for Defence Medicine (Academia and Research), Birmingham, United Kingdom; b CBR Division, Defence Science and Technology Laboratory (Dstl), Porton Down, Salisbury, Wiltshire, United Kingdom; c Public Health England, Porton Down, Salisbury, Wiltshire, United Kingdom; d College of Life and Environmental Sciences, University of Exeter, Exeter, United Kingdom

**Keywords:** *Coxiella*, Q fever, antibiotic, treatment

## Abstract

Q fever, caused by the intracellular pathogen Coxiella burnetii, is traditionally treated using tetracycline antibiotics, such as doxycycline. Doxycycline is often poorly tolerated, and antibiotic-resistant strains have been isolated. In this study, we have evaluated a panel of antibiotics (doxycycline, ciprofloxacin, levofloxacin, and co-trimoxazole) against C. burnetii using *in vitro* methods (determination of MIC using liquid and solid media; efficacy assessment in a THP cell infection model) and *in vivo* methods (wax moth larvae and mouse models of infection). In addition, the schedule for antibiotic treatment has been evaluated, with therapy initiated at 24 h pre- or postchallenge. Both doxycycline and levofloxacin limited overt clinical signs during treatment in the AJ mouse model of aerosol infection, but further studies are required to investigate the possibility of disease relapse or incomplete bacterial clearance after the antibiotics are stopped. Levofloxacin was well tolerated and therefore warrants further investigation as an alternative to the current recommended treatment with doxycycline.

## INTRODUCTION

Coxiella burnetii is an obligate intracellular bacterium that causes Q fever. It is asymptomatic in up to 60% of infections (1) and otherwise typically presents as an acute undifferentiated febrile illness, which is normally self-resolving after a few weeks (2). However, Q fever can manifest in significant long-term health issues; chronic fatigue occurs in up to 20% of acute cases (3); and in an estimated 1% to 5% of cases, chronic Q fever can develop, resulting in conditions such as endocarditis associated with significant mortality (4). C. burnetii is a zoonotic infection with reservoirs in ruminant animals, such as goat and cattle, and there are reports of outbreaks of infections acquired downwind of infected farms (5). C. burnetii has the ability to form a spore-like structure (termed small cell variant) with good environmental stability especially in dry, dusty atmospheres (6). These factors have combined to present a problem for British Military soldiers working in places like Afghanistan (7), which is further compounded by the complexities of confirming a diagnosis.

The first-line therapy for acute Q fever is doxycycline (200 mg/day) for 14 days. Tetracycline administration within the first 3 days of illness has been shown to reduce the duration of fever by up to 50% (8). Doxycycline in combination with hydroxychloroquine in excess of 18 months is the standard antibiotic regimen for chronic disease (9). As doxycycline can be poorly tolerated, with up to 23% of patients reporting adverse effects, including gastritis, candidiasis, and occasionally severe photosensitivity (10), there is a need to explore other treatment options. It has also been postulated that because 100 mg of doxycycline daily is accepted as a form of malaria prophylaxis and is reasonably well tolerated, it could also be beneficial as a prophylaxis against Q fever (7). Preventing infection would be of benefit, as asymptomatic infections can also develop into chronic Q fever (3).

The antibiotics doxycycline, moxifloxacin, and other fluoroquinolones have been shown, by a retrospective analysis during the Netherlands outbreak, to lower the rate of hospitalization compared with other treatments, such as a beta-lactam or azithromycin (11). Co-trimoxazole is the recommended treatment for pregnant women. These antibiotics are readily available to the military on deployment and exercise. We and others have explored the efficacy of these alternative antibiotics against C. burnetii in the axenic growth medium ACCM-2 (12–14). However, for an obligate intracellular bacterium, it is important to assess the efficacy of each antibiotic within the intracellular environment. Such an assessment is important because the cell also independently exerts a bactericidal effect with the release of reactive oxygen intermediates, nitric oxide, and cytokines (15). Therefore, intracellular growth assays allow the combined effect of antibiotic efficacy, intracellular uptake and localization of drug and bacteria, and cellular bactericidal effects to be investigated. The contribution of the host immune system also requires evaluation. Currently, reports of researchers assessing antibiotic efficacy in well-characterized animal models are very limited (16).

Therefore, we aimed to evaluate the efficacy of alternative, potentially better tolerated antibiotics to treat Q fever by comparing *in vitro* and *in vivo* methods from broth growth and *in vitro* intracellular cell assays, through a lower species *in vivo* infection model (Galleria mellonella wax moth larvae) (17) and the well characterized AJ mouse model of acute Q fever (18, 19). Furthermore, we have also explored the utility of these alternative antibiotics as a prophylaxis to inform future treatment strategies for patients with acute Q fever.

## RESULTS

### Determination of MICs of doxycycline, levofloxacin, ciprofloxacin, or co-trimoxazole against C. burnetii using axenic media.

Previously, MICs have been determined against C. burnetii NMII (12); in this study, the MICs have been extended to include C. burnetii NMI (Table 1). Doxycycline hyclate exhibited the lowest MIC against NMI (0.01 to 0.04 μg/ml) and co-trimoxazole exhibited the highest MIC (8 to 32 μg/ml). The MIC for levofloxacin was 0.5 to 1 μg/ml and for ciprofloxacin was 1 to 4 μg/ml. These results are consistent with the MICs established against NMII. The minimal bactericidal concentrations (MBCs) were 8 μg/ml for doxycycline hyclate, and the remaining antibiotics had an MBC greater than the highest concentration tested for the MICs (levofloxacin, >16 μg/ml; ciprofloxacin, >8 μg/ml; and co-trimoxazole, >128 μg/ml) (Table 1). In addition, a statistically significant inhibition of bacterial growth was assessed by comparing the bacterial growth at 7 days for each antibiotic concentration in relation to the bacterial growth at 7 days in the absence of antibiotics to enable comparison to the THP-1 data. Doxycycline hyclate had significant inhibitory effects at concentrations of >0.01 μg/ml (*P* < 0.001), levofloxacin at concentrations of >0.5 μg/ml (*P* < 0.01), ciprofloxacin at concentrations of >0.5 μg/ml (*P* < 0.05), and co-trimoxazole at concentrations of >1 μg/ml (*P* < 0.05) (Table 1).

**TABLE 1:** Antibiotic efficacy for C. burnetii NMI^*a*^

Antibiotic	MIC NMII^*b*^	MIC NMI^*b*^	MIC NMI^*c*^	MBC NMI^*c*^	THP-1 NMI^*d*^
Doxycycline hyclate	0.04–0.16	0.01–0.04	0.01	8	0.2
Levofloxacin	0.5–4	0.5–1	1	>16	0.16
Ciprofloxacin	1–2	1–4	1	>16	0.5
Co-trimoxazole	16–32	8–32	8	>128	2

a MIC was defined as the lowest concentration of antibiotic required to inhibit bacterial growth in 6-day cultures; MBC was defined as the lowest concentration to give no visible growth when plated onto ACCM-2 agar and incubated for 7 days. All values are μg/ml.

b Determined by OD in broth culture. MIC NMII is taken from previously published work (12).

c Determined by CFU in broth culture.

d Significant inhibition of growth in THP-1 cells was determined at 72 h postinfection, compared with growth in 0 μg/ml antibiotic, using a one-way ANOVA with Dunnett’s multiple comparisons.

### Evaluation of antibiotic efficacy against C. burnetii in a THP-1 model of infection.

In order to determine the efficacy of antibiotics within an intracellular environment, a THP-1 cell infection assay was used. The range of antibiotic concentrations was selected from the MIC via broth dilution results (Table 1). A statistically significant inhibition of bacterial growth was assessed by comparing the bacterial growth at 72 h for each antibiotic concentration in relation to the bacterial growth at 72 h in the absence of antibiotics. Doxycycline at concentrations of >0.2 μg/ml significantly inhibited growth in comparison to growth in the absence of antibiotics (*P *< 0.01), levofloxacin displayed a significant inhibition of intracellular bacterial growth at concentrations of >0.16 μg/ml (*P *= 0.001), ciprofloxacin demonstrated a significant inhibition of bacterial growth at concentrations of >0.5 μg/ml (*P *< 0.01), and co-trimoxazole at concentrations of >2 μg/ml demonstrated significant inhibitory effects of bacterial growth (*P *< 0.05).

### *In vivo* antibiotic efficacy in a G. mellonella model.

**FIG 1:**
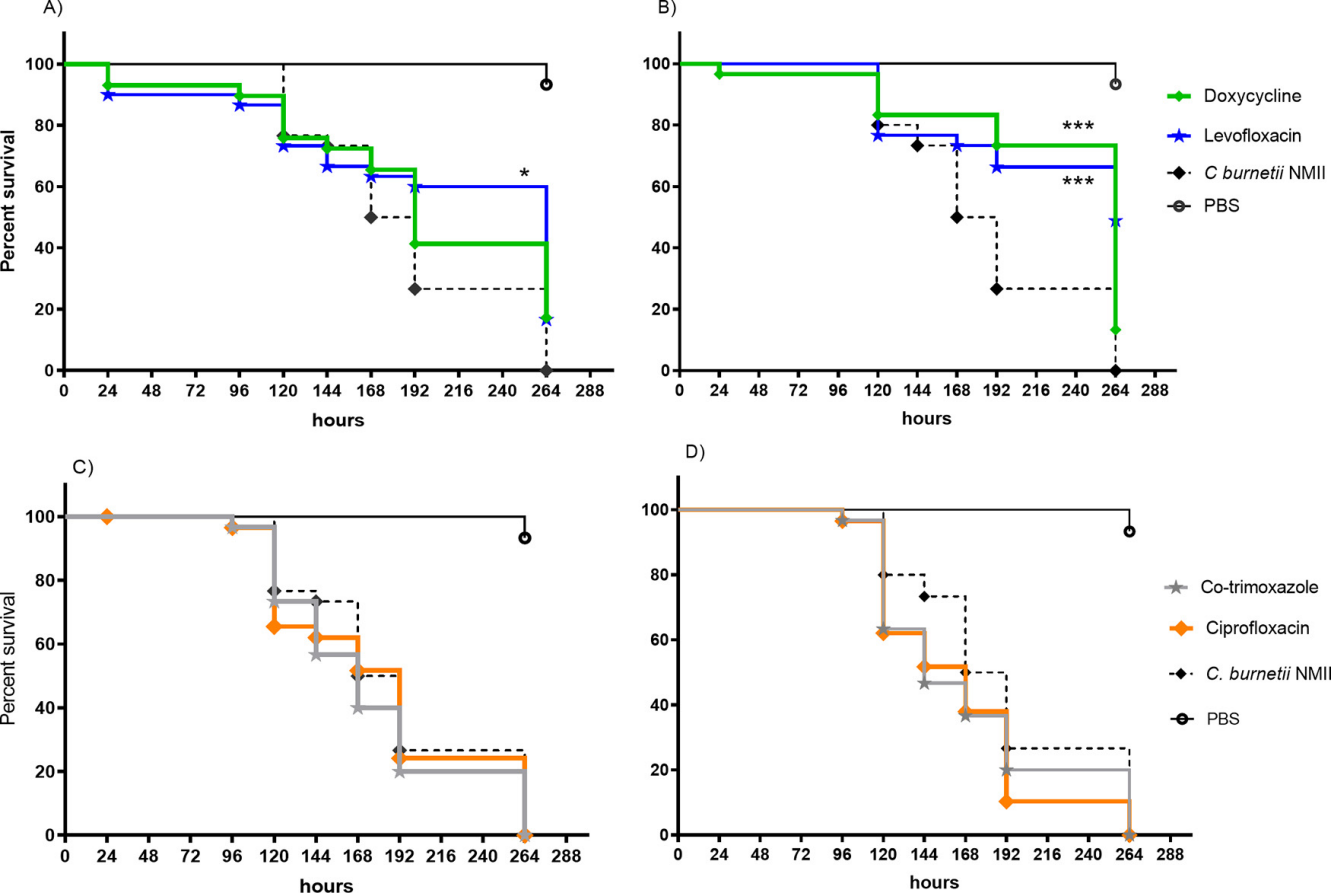
Survival of G. mellonella following challenge with 1 × 10^6^ GE of C. burnetii NMII and treated with 50 mg/kg of either PBS (solid black line), levofloxacin (blue line), or doxycycline (green line) 24 h prechallenge (A) or postchallenge (B) and co-trimoxazole (purple line) or ciprofloxacin (orange line) 24 h prechallenge (C) or postchallenge (D). Significance by log-rank (Mantel-Cox) test is shown; *, *P *< 0.05; ***, *P *< 0.001.

In order to determine the antibiotic efficacy within an *in vivo* system, doxycycline, levofloxacin, ciprofloxacin, and co-trimoxazole (all at 50 mg/kg of body weight, and more than 10-fold greater than the determined MIC) were administered to G. mellonella larvae as a single dose either 24 h pre- or postchallenge with C. burnetii NMII at 1 × 10^6^ genome equivalents (GE). The survival of the larvae was recorded over 264 h, and the median time to death determined. Levofloxacin treatment prechallenge significantly extended the time to death (264 h versus 180 h, *P *< 0.05), whereas doxycycline did not (Fig. 1A); however, both levofloxacin and doxycycline given postchallenge significantly extended the median time to death to greater than 264 h (*P *< 0.001) (Fig. 1B). Neither ciprofloxacin nor co-trimoxazole treatment pre- or postchallenge resulted in extended time to death (Fig. 1C and D). Less than 10% of G. mellonella died within the control groups of either phosphate-buffered saline (PBS) or antibiotic alone, and therefore, time to death was not calculable.

### Doxycycline, levofloxacin, ciprofloxacin, sulfamethoxazole, and trimethoprim pharmacokinetics (PKs) in A/J mice.

**FIG 2:**
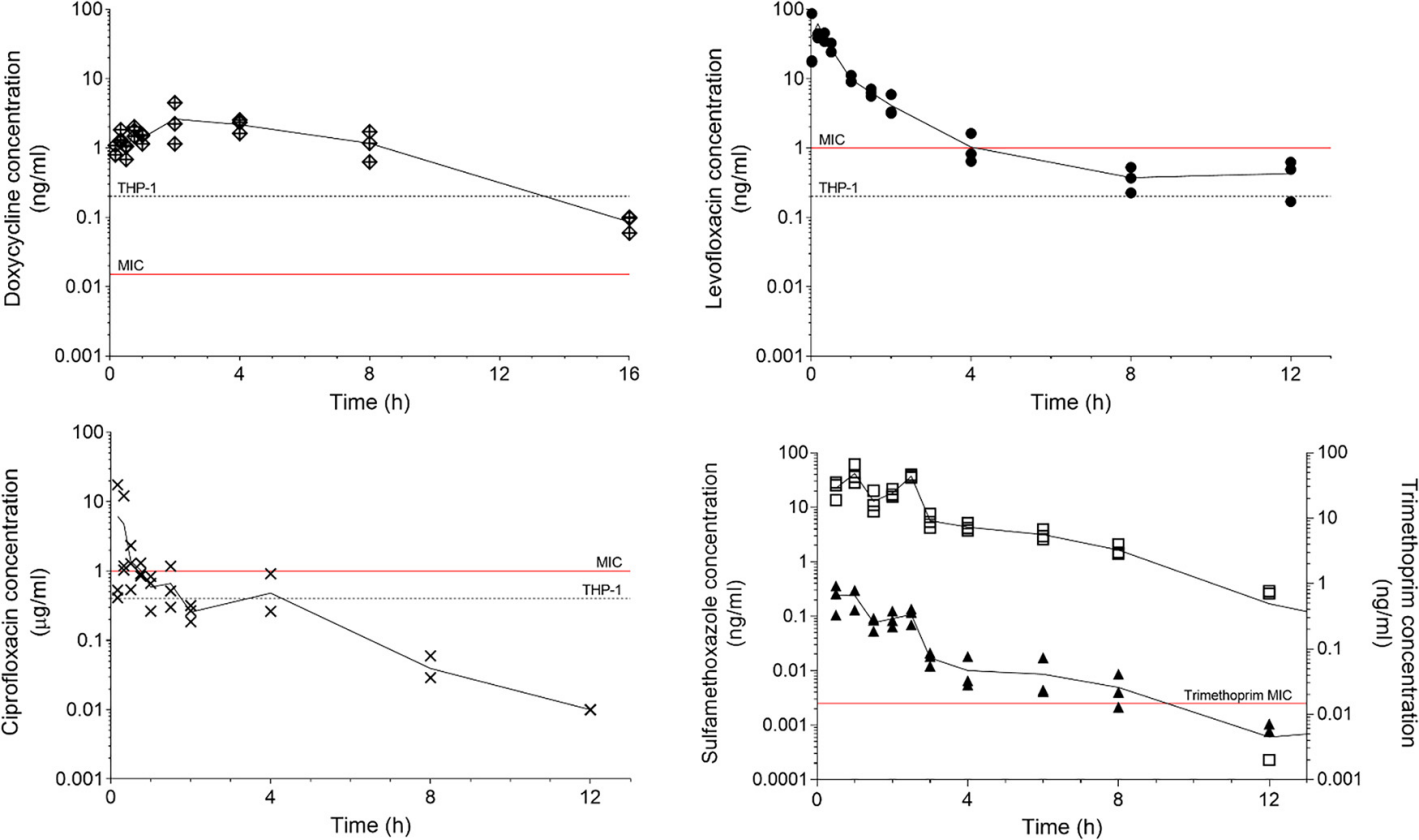
The efficiency of drug eradication from mouse serum over time following a single dose of doxycycline (50 mg/kg), levofloxacin (100 mg/kg), ciprofloxacin (50 mg), and co-trimoxazole (48 mg/kg; consists of 40 mg/kg sulfamethoxazole and 8 mg/kg trimethoprim). The MIC determined from growth in ACCM-2 broth and the significant inhibitory concentration determined in the cell based THP-1 assay are marked.

The concentration-time profiles for doxycycline (50 mg/kg), levofloxacin (100 mg/kg), ciprofloxacin (50 mg/kg), and sulfamethoxazole and trimethoprim (48 mg/kg co-trimoxazole consisting of 40 mg/kg sulfamethoxazole and 8 mg/kg trimethoprim) were determined and the pharmacokinetic parameters estimated by noncompartmental analysis (Fig. 2). The pharmacokinetically guided approach (18) was used to calculate the human equivalent murine dose for use in subsequent efficacy studies (Table 2). The human doses used as targets were those used clinically to treat Q fever (19–21), with the exception of co-trimoxazole, which is a dual formulation of sulfamethoxazole and trimethoprim. There are no published human pharmacokinetic data for co-trimoxazole; therefore, the target dose was taken as above the MIC for two-thirds of the time (22).

**TABLE 2:** Pharmacokinetic parameters in AJ mice for antibiotics, calculated from the data shown in Fig. 2^*a*^

Antibiotic	Route	*t*_1/2_ (h)	AUC_0–t_ (μg·h/ml)	CL (l/h/kg)	Target AUC (μg·h/ml)	Human treatment regimen (mg)	Murine dose (mg/kg)
Doxycycline hyclate	Oral	3.5	21.3	2.6	40–123	100 bd	105 bd
Levofloxacin	Intraperitoneal	2.4	48.6	1.7	45.6	500 od	40 bd
Ciprofloxacin	Intraperitoneal	1.6	15.6	1.9	23.2	500 bd	22 bd
Sulphamethoxazole	Oral	1.9	123.5	0.3	>2 μg/ml, ¾ time	960 od	40 bd (48 bd co-trimoxazole)
Trimethoprim	Oral	1.6	0.4	18.7		960 od	8 bd (48 bd co-trimoxazole)

a *t*_1/2_, half-life; AUC_0–t_, area under the concentration-time curve from 0 to t; CL, clearance; bd, twice a day; od, once a day.

### *In vivo* antibiotic efficacy in a murine model.

**FIG 3:**
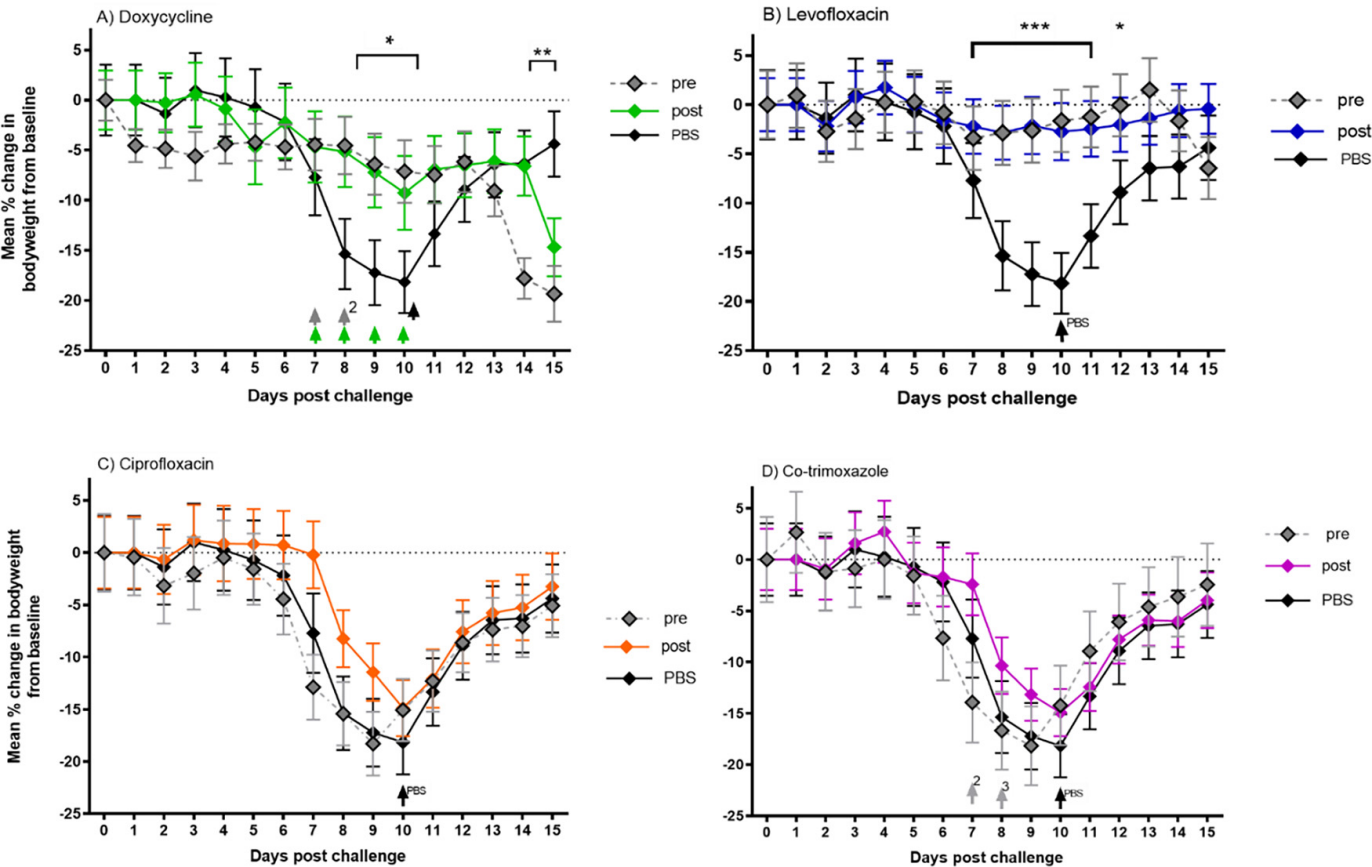
Mean percentage change in body weight of mice as a measurement of efficacy of 105 mg/kg oral doxycycline (A), 40 mg/kg intraperitoneal levofloxacin (B), 22 mg/kg intraperitoneal ciprofloxacin (C), and 48 mg/kg oral co-trimoxazole (D); all given twice daily and commenced at 24 h prechallenge (gray diamonds) or 24 h postchallenge (colored diamonds) with C. burnetii. Mice were challenged inhalationally with 1 × 10^7^ GE/ml of C. burnetii. Significant differences from PBS-treated control mice (black diamonds) are marked. *, *P *< 0.05; **, *P* < 0.01; ***, *P *< 0.001; significance determined by ANOVA. Arrows denote mice culled on welfare grounds.

A/J mice were challenged with a mean presented dose of 1 × 10^7^ GE C. burnetii via the aerosol route and treated with oral doxycycline hyclate (105 mg/kg twice daily), intraperitoneal (i.p.) levofloxacin (40 mg/kg twice daily), intraperitoneal ciprofloxacin (22 mg/kg twice daily), and oral co-trimoxazole (48 mg/kg twice daily) for 7 consecutive days after challenge, starting either 24 h pre- or 24 h postchallenge. The mean weight losses for the groups for 15 days after challenge are displayed in Fig. 3. Mice treated with PBS reached peak weight loss by day 9 (18%) and 80% exhibited clinical signs, such as ruffled fur and an arched back at least once from days 7 to 10 postchallenge.

Doxycycline commenced pre- and postchallenge significantly reduced weight loss (Fig. 3A) (*P* < 0.05), which peaked in the prechallenge group at day 11 (7%) and day 10 (9%) in the postchallenge group. Milder clinical signs were seen in the postchallenge group, with 50% exhibiting ruffled fur at least once from day 6 to 10. Early onset weight loss was seen in the prechallenge group that corresponds to clinical signs appearing at least once in 60% of mice from day 1 to day 9 (ruffled fur and arched back). These early adverse signs appear to be a drug side effect, as they emerge earlier than either weight loss or clinical signs in the PBS-treated group. Four animals from the doxycycline pretreatment group and three from the posttreatment group were culled on welfare grounds, compared with one from the control group (marked by arrows, Fig. 3A).

Levofloxacin pre- and postchallenge significantly reduced weight loss (Fig. 3B) (*P *< 0.001), with a peak weight loss in the prechallenge group at day 7 (3%) and in the postchallenge group at day 10 (3%). Neither of these groups showed adverse clinical signs, and all animals survived. Treatment with ciprofloxacin and co-trimoxazole pre- and postchallenge did not reduce weight loss or clinical signs compared with PBS-treated mice (Fig. 3C and D). Pretreatment with co-trimoxazole was highly detrimental, and 5 animals were culled on welfare grounds. Both doxycycline and the levofloxacin prechallenge group show signs of weight loss at the end of the study, which is suggestive of delayed disease (significant for the doxycycline treated groups, *P* value of <0.01 for prechallenge and *P* value of <0.05 for postchallenge).

**FIG 4:**
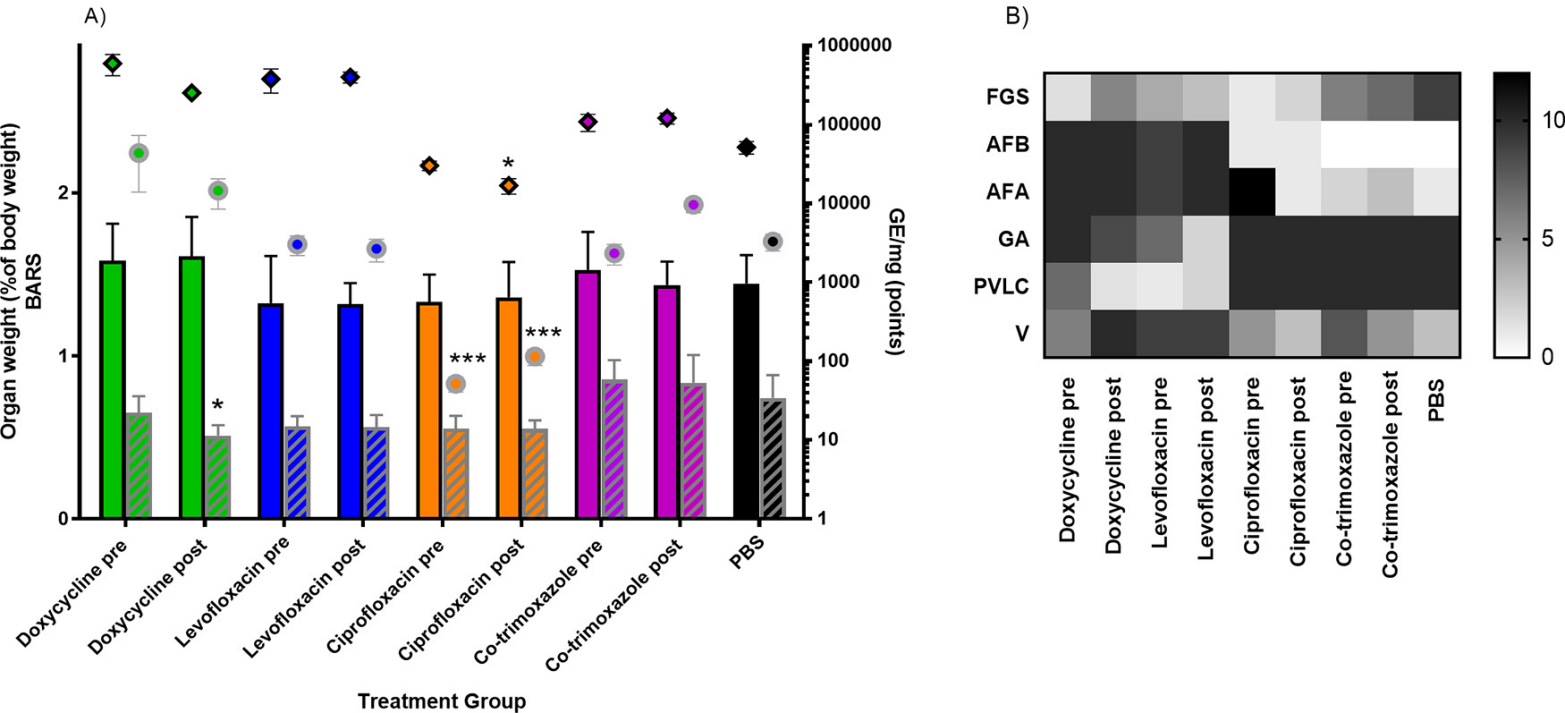
Physiological features of disease in mice 14 days postchallenge treated twice daily with 105 mg/kg doxycycline, 40 mg/kg levofloxacin, 22 mg/kg ciprofloxacin, or 48 mg/kg co-trimoxazole, commenced 24 h pre- or 24 h postchallenge for 7 days postchallenge and PBS-treated control. Mice were challenged by inhalation with 1 × 10^7^ GE/ml of C. burnetii. (A) Weight of lungs (colored bars) and spleens (striped bars) as a percentage of body weight as mean and SE and the corresponding bacterial colonization of lung (black outlined diamonds) and spleen (gray outlined circles) as determined by qPCR (mean and SE). Significant differences from the PBS-treated control group are indicated as follows: *, *P *< 0.05; **, *P* < 0.01; ***, *P *< 0.001. (B) Occurrence of the histological features associated with Q fever at day 14 pc (regardless of severity). Features are focal granulomatous splenitis (FGS), acute focal bronchiolitis (AFB), acute focal alveolitis (AFA), granulomatous alveolitis (GA), perivascular lymphoid cuffs (PVLC), and vasculitis (V).

Infection with C. burnetii is known to significantly increase the weight of lungs and spleen in untreated mice. Treatment with doxycycline postchallenge significantly moderated the spleen weight increase (Fig. 4A) (*P*< 0.05), while all other treatment groups showed no significant moderation to organ weight at the end of the study. In addition, mice treated with PBS were colonized with approximately 2 × 10^4^ to 1 × 10^5^ GE/ml in their lung and 1 × 10^3^ to 7 × 10^3^ GE/ml in their spleen on day 14 postchallenge (Fig. 4A). Treatment with ciprofloxacin both pre- and postchallenge significantly reduced the bacterial burden in the spleen resulting in a 1- to 2-log drop in bacterial burden by quantitative PCR (qPCR) (*P* < 0.001), and ciprofloxacin postchallenge also led to a 0.5-log reduction in bacterial burden in the lung (*P* < 0.05). There was no reduction in burden in any other group; pre- and posttreatment with doxycycline, levofloxacin or co-trimoxazole resulted in higher counts in the lung samples on day 14.

Histological examination of the lung and spleen samples determined that on day 14 postchallenge, the levofloxacin- and doxycycline-treated groups (both pre- and postchallenge) had a different prevalence of lesions than the PBS control and co-trimoxazole- and ciprofloxacin-treated groups (Fig. 4B). For the levofloxacin and doxycycline groups, the C. burnetii-associated features were most commonly acute lung damage with less splenic involvement, which is characteristic of early stages of the disease. The PBS, ciprofloxacin, and co-trimoxazole groups have greater proportions of granulomatous changes which are associated with disease resolution (23). There is the least splenic involvement in the ciprofloxacin groups which corresponds to the reduced bacterial load quantified by PCR (Fig. 4B), which suggests that in these groups, the systemic spread was limited or the damage was less severe.

## DISCUSSION

This is the first study using different *in vitro* and *in vivo* methods to determine the efficacy of doxycycline, levofloxacin, ciprofloxacin, and co-trimoxazole to treat Q fever. Doxycycline for 2 weeks is the standard treatment for C. burnetii in humans (13); however, it is important to assess alternative antibiotic options where doxycycline contraindicated cannot be tolerated or where resistant strains are isolated. The sensitivity to the selected antibiotics was similar for both virulent phase I C. burnetii and avirulent phase II C. burnetii using *in vitro* methods, despite phase II bacteria lacking a full-length O-antigen (24). Using a broth dilution method, MIC results for all antibiotics were comparable to those derived from shell vial assays and cell culture (22, 25, 26). In addition, doxycycline has the lowest MIC (0.01 μg/ml) compared with the antibiotics tested in this study.

Antibiotic efficacy in broth is a useful initial screen for novel antibiotics, as simpler systems are easier to interpret and may be more reproducible, allowing a direct assessment of the antimicrobial effect. However, C. burnetii resides entirely intracellularly and so cellular uptake of antibiotics, co-localization of drug and bacteria, and cellular bactericidal effects are all important factors that need to be considered. Therefore, human monocytic THP-1 cells were used in this study because they can be differentiated into functioning macrophages, the primary target of C. burnetii, allowing human macrophage immune functions, such as the release of reactive oxygen intermediates, nitric oxide, and cytokines to be combined with assessing antibiotic efficacy (27–29).

Doxycycline had enhanced efficacy in broth compared with the THP-1 assay where, in the intracellular model, doxycycline was not bactericidal at 8 μg/ml (data not shown). Doxycycline is bacteriostatic against C. burnetii and is only bactericidal when combined with hydroxychloroquine in chronic infection (13). Hydroxychloroquine increases the pH in the lysosome which is the postulated mechanism for allowing doxycycline to exert a bactericidal effect (14). However, as the ACCM medium is also acidic, the difference between broth and intracellular doxycycline efficacy may be due to poor penetration or retention of the antibiotic within the parasitic vacuole within the cell and may be assessed in the future using intracellular PK or uptake studies, with the addition of hydroxychloroquine. Both ciprofloxacin and levofloxacin had an MIC of 1 μg/ml that was determined using broth dilution, and both maintained activity intracellularly, with a bactericidal effect observed in broth or THP-1 cells at concentrations of >2 μg/ml of antibiotic (data not shown). These data are comparable to previous data (30). Quinolones accumulate within neutrophils and macrophages which may account for increased activity against intracellular C. burnetii (31). However, this may be dependent on the cell type, with the presence Mrp4, a ciprofloxacin efflux transporter, in J774 mouse macrophage cells, whereas no active efflux is observed in THP-1 cells (32). Co-trimoxazole is used in the treatment of Q fever during pregnancy or where doxycycline is contraindicated (33), although retrospective analyses of clinical outcomes are mixed (34). In this study, the MIC determined using broth dilution is high (8 to 32 μg/ml), but this may be because, unlike the other antibiotics tested, co-trimoxazole is unstable in ACCM for 7 days (data not shown). However, similar low susceptibility *in vitro* has been observed using qPCR or immunofluorescence (22). Despite poor activity in broth, inhibition of intracellular growth was observed which may account for clinical outcomes.

While all the antibiotics evaluated were shown to have activity against C. burnetii in the *in vitro* assays, this was not replicated in the G. mellonella model. Only doxycycline given at 24 h postchallenge or levofloxacin given 24 h pre- or postchallenge were shown to have a significant survival benefit. Although the larvae received only a single dose at 50 mg/kg (equivalent to 50 μg/ml), this concentration exceeds the broth MBC for both ciprofloxacin and doxycycline by at least 3-fold and was 25 times above the intracellular inhibitory concentration determined in the THP-1 assay for all antibiotics tested. Ciprofloxacin was predicted to have been as protective as levofloxacin in this model, as the half-life of ciprofloxacin in G. mellonella has been determined to exceed 15 h compared with 2 h for levofloxacin (35, 36), but ciprofloxacin provided no protection. Equally, doxycycline would have been predicted to provide some protection as a pretreatment, its effective intracellular concentration (determined in the THP-1 assay) is similar to levofloxacin, and its half-life in G. mellonella of 17 hours is considerably longer (36). Further pharmacokinetic research in the larvae to find optimum timing for pre- and postchallenge antibiotics is required for C. burnetii to inform future efficacy studies.

In this study, mouse PK data were used to determine a dose equivalent to that currently used to treat human cases of Q fever (11). Understanding the target concentrations required to control C. burnetii in intracellular assays gives us an additional perspective on the efficacy of each of the antibiotics. Doxycycline persisted in mouse plasma above the MIC and above the efficacious level determined in the THP-1 intracellular assay for the entire treatment period. Doxycycline treatment initiated prechallenge as a prophylaxis and postchallenge as a treatment significantly reduced weight loss compared with the PBS control at the peak of disease, but both groups appeared to relapse by the end of the study with weight loss. The same result was seen with levofloxacin pre-exposure which significantly reduced weight loss at the peak of disease compared with the PBS control, but again, the mice started to lose weight by the end of the study. This finding supports the theory that, while doxycycline may prevent bacterial replication, it is the immune system that provides clearance and that treating the disease too soon and controlling signs and symptoms may not be beneficial in eradication of this intracellular bacterium in the longer term (37). It may be that extended therapy to 14 days would improve outcomes, and future work to evaluate efficacy when antibiotics are initiated later in the incubation period are required. It is likely that the weight loss at the end of the mouse study and the relatively high levels of bacterial load recorded for the tissue samples from the animals in these treatment groups are due to delayed disease due to the bacteriostatic and not bactericidal effect of the antibiotics. Bacterial load assessed by viable count rather than qPCR would have been more informative to accurately measure clearance in tissues (38).

Groups treated with ciprofloxacin pre- and postchallenge had no weight loss reduction compared with the PBS control, which supports previous observations (39). This result was predicted from the PK mouse data; using the human equivalent dose in the mouse study resulted in the concentration in the mouse sera being above the inhibitory intracellular concentration (determined in the THP-1 assay) for only 1 hour. It is therefore intriguing that, in terms of preventing systemic spread of disease and protecting the spleen from C. burnetii associated damage, ciprofloxacin was the most effective treatment.

Co-trimoxazole has limited efficacy against C. burnetii in ACCM-2 broth, G. mellonella, and AJ mice, where no protection against weight loss, splenomegaly, or bacterial colonization was seen. While there are limitations to all *in vitro* and *in vivo* models, observational human studies of the benefits of co-trimoxazole in pregnancy have shown conflicting results, and the efficacy of co-trimoxazole in Q fever remains unclear (34, 40, 41).

Although there was some evidence of levofloxacin being an effective as prophylaxis in the G. mellonella model, in the mouse model, there was no evidence of a prophylactic effect for any of the antibiotics tested. We postulate this was partly due to antibiotic immunomodulation being disadvantageous when used as prevention or early in the treatment for Q fever. Tetracyclines (e.g., doxycycline) and fluoroquinolones (levofloxacin and ciprofloxacin) regulate the expression of Toll-like receptors (TLRs) and cytokines which manifests as a reduction in proinflammatory cytokines, such as interleukin-1 (IL-1), IL-6, and tumor necrosis factor alpha (TNF-α) (42–44). Doxycycline but not ciprofloxacin has been shown to reduce the production of interferon gamma (IFN-γ) and lipopolysaccharide (LPS)-induced nitric oxide production (45). These factors are important in controlling replication of C. burnetii and formation of the parasitophorous vacuole (46).

We report the evaluation of antibiotics used to treat Q fever using axenic broth dilution, THP-1 cell infection, G. mellonella, and inhalational AJ mouse models. The comparison of efficacy from *in vitro* and *in vivo* methods highlights the limitations around extrapolation of antibiotic efficacy using *in vitro* methods alone. For example, co-trimoxazole appeared to perform better in a cell-based assay than in broth dilution but failed to be efficacious in the mouse model. Similarly, ciprofloxacin was efficacious *in vitro* but not *in vivo.* However, both levofloxacin and doxycycline had a low MIC in broth, demonstrated significant intracellular inhibition, and showed some ability to control infection in G. mellonella and mice. Levofloxacin showed potential as an alternative treatment to the standard doxycycline and was effective and better tolerated by the mouse. Treatment by either antibiotic in the mouse model suggested a delay in disease rather than a cure. Future work will extend the study timescale using the murine model to further evaluate disease progression, clearance, and alternative regimes to treat acute Q fever. This information may be included in future human treatment strategies, including alternative antibiotics or dosing regimens.

## MATERIALS AND METHODS

### Bacteria.

C. burnetii NMI (the infectious phase I variant, RSA493) was used in the murine model and for the cell assays, and C. burnetii NMII (the avirulent phase II variant, RSA439, clone 4) was used in the G. mellonella model. C. burnetii was cultured axenically in ACCM-2 (Sunrise Science Products, San Diego, CA) and incubated statically at 37°C (in 5% CO_2_ and 2.5% 0_2_) for 7 days or shaken at 75 rpm in a sealed container with a GENbox microaer atmosphere generator (bioMérieux, France) (for animal challenge). The bacteria were centrifuged at 10,000 × *g* for 20 min, resuspended in PBS at a concentration of approximately 1 × 10^9^ genome equivalents (GE)/ml, and stored frozen. All manipulations of C. burnetii were carried out in a class III microbiological safety cabinet complying with British Standard EN12469:2000. All studies were risk assessed and approved by Dstl’s Biosafety Committee.

### Bacterial enumeration by PCR.

C. burnetii was enumerated using quantitative PCR (qPCR) using the methodology in Norville et al. (39). qPCR targeted the *sod* gene using the forward primer TCTTCAACAATGCAGCACAACAT and reverse primer TGAAGCCAATTCGCCAGAA. The probe sequence was CATTTTATTGGCACTGCATGAGCCCTG, and the probe was covalently labeled at the 5′ end with the reporter dye 6-carboxyfluorescein (FAM) and at the 3′ end with the quencher dye black hole quencher 1 (BHQ-1). Chromosomal DNA was extracted by addition using the Qiagen QIAmp DNA mini, blood mini, or tissue kit depending on the tissue type. Next, 2-μl template DNA, forward primer (900 nM), reverse primer (900 nM), probe (250 nM), and ABI Fast TaqMan master mix were used. PCR cycles consisted of 3 min at 95°C and 30 s at 60°C, followed by 50 2-step cycles, with 1 cycle consisting of 15 s at 95°C and 30 s at 60°C. For each PCR, a control of linearized synthetic plasmid was quantitated after linearization and purification using an ND-2500 NanoDrop spectrophotometer. A standard curve of the synthetic plasmid was run in duplicate in the range of 1 × 10^7^ GE/ml to 1 × 10^2^ GE/ml for each PCR. The lower limit of detectection for this assay is 4.4 × 10^1^ GE/mg for spleen or 2.2 × 10^1^ GE/mg for lung samples.

### Bacterial enumeration by CFU.

For the THP-1 cell-based assay and broth assay, bacteria were enumerated by serial dilution in PBS, plated onto ACCM-2 agar, and incubated at 37°C (in 5% CO_2_ and 2.5% O_2_) for 10 days.

### Antimicrobial agents.

Doxycycline hyclate powder and levofloxacin powder (Sigma-Aldrich, United Kingdom) were diluted in distilled water (DiH_2_O), ciprofloxacin powder (Sigma-Aldrich) was diluted in DiH_2_O, and 1 M sodium hydroxide and 100 mg/ml co-trimoxazole liquid (Sigma-Aldrich) were diluted in dimethyl sulfoxide (DMSO) to a working concentration of 10 mg/ml for use in the cell culture assays and the G. mellonella model. Doxycycline hyclate powder (Sigma-Aldrich) was diluted in DiH_2_O, a 2-mg/ml solution of ciprofloxacin (Hospira UK Ltd., Horizon, Maidenhead, UK) was used neat, a 5-mg/ml solution of levofloxacin (Hospira UK Ltd., Horizon) was used neat, and 40 mg/200 mg of co-trimoxazole per 5-ml pediatric solution (Aspen Pharma Trading Limited, Dublin, Ireland) was diluted in PBS to the required concentrations for the A/J mouse study.

### Cells.

THP-1 cells were sourced from European Collection of Authenticated Cell Cultures (ECACC; PHE, Porton Down, UK). Cells were maintained in 75-cm^3^ flasks in loose suspension at a cell density greater than 1 × 10^5^ cells/ml in RPMI 1640 (Life Technologies, Gaithersburg, MD) plus 2 mM l-glutamine and 10% fetal calf serum in 5% CO_2_ at 37°C.

### Determination of the MIC and minimum bactericidal concentration (MBC).

Results for NMII MIC were taken from Clay et al. (12) and had been determined as the lowest concentration of an antimicrobial agent that prevented visible growth (measured via optical density [OD] at 590 nm) in a broth dilution susceptibility test (in accordance with the Clinical and Laboratory Standard Institute guidelines). Briefly, a range of increasingly concentrated antibiotic broths were made, inoculated with approximately 5 × 10^6^/ml (final concentration) of culture, and then incubated for 6 days (in 5% CO_2_ and 2.5% O_2_) when the OD was measured. The MIC for NMI was determined both by measuring the OD and then also plating out each broth to obtain a viable bacterial count to make it comparable to the THP-1 intracellular assay. The MBC was determined by plating out at the neat broth onto ACCM-2 plates, and no visible bacterial growth appeared following incubation after 10 days. Significant inhibition of bacterial growth compared with the bacterial count after 7 days in the presence of no antibiotics (0 μg/ml) was assessed using a one-way analysis of variance (ANOVA) with Dunnett’s multiple comparisons. All tests were done in triplicate.

### Cell assay.

THP-1 cells were seeded at 5 × 10^5^ cells/ml into a 24-well plate in RPMI 1640 (Life Technologies, Gaithersburg, MD) containing 10% fetal calf serum (FCS) + 2 mM glutamine with 200 nM phorbol myristate acetate (PMA) (Sigma-Aldrich, UK) to stimulate differentiation into the macrophage-like cells and incubated at 37°C and 5% CO_2_ for 72 hours (47). Following incubation, cells were assessed for viability using trypan blue staining. Phase I C. burnetii stock was diluted in Leibovitz’s L15 medium with 10% FCS (Life Technologies). Cells were infected with a multiplicity of infection (MOI) of 20:1 (time [T], −24 h), and bacteria were allowed to infect for 24 hours. Antibiotic stocks were diluted to a range of concentrations in L15 plus 10% FCS. At 24 hours after cell infection (designated T, 0 h), the cells were washed twice with PBS and 1 ml of antibiotic media was added to duplicate wells. At Ts of 0 h and 72 h, pairs of wells were lysed with diH_2_0, pooled, serially diluted, and plated to allow enumeration of intracellular bacteria. Each experiment was completed in triplicate.

### Wax moth larvae.

Galleria mellonella was sourced from Live Foods UK and maintained on wood chips at 14°C. Healthy larvae weighing between 0.19 and 0.26 g were selected. The model of infection was set up as described by Norville et al. (39). Groups of 10 larvae were injected with 1 × 10^6^ GE/ml C. burnetii NMII in 10 μl into their uppermost right proleg. The larvae were treated with a range of antibiotics into their left uppermost proleg given once, either 24 h pre- or 24 h postchallenge. Untreated larvae were injected with PBS at 24 h postchallenge. The study was completed in triplicate. Groups of 5 uninfected control larvae were treated with either PBS, doxycycline, levofloxacin, ciprofloxacin, or co-trimoxazole to ensure there were no adverse effects from the antibiotics alone.

### Mice.

Groups of age-matched AJ mice (Envigo, Bicester, UK) were housed on a 12-h day-night light cycle, with food and water available *ad libitum* in an Advisory Committee on Dangerous Pathogens (ACDP) (UK) level 3 flexible-film isolator and allowed to acclimatize before challenge. All procedures were conducted under a project license approved by internal ethical review and in accordance with both the UK Animal (Scientific Procedures) Act (1986) and the 1989 Codes of Practice for the Housing and Care of Animals used in Scientific Procedures

### Pharmacokinetic studies.

Pharmacokinetic (PK) data for intraperitoneal ciprofloxacin and oral doxycycline were taken from Norville et al. (39) and remodeled for the purposes of this study. Levofloxacin was administered to mice (*n* = 30) via the i.p. route at a dose of 100 mg/kg. Co-trimoxazole was administered to 30 mice orally via pipette at a dose of 48 mg/kg. Groups of 3 mice were culled at 1, 10, 20, and 30 min and 1, 1.5, 2, 4, 8, and 12 h after dosing. Blood samples were collected from all animals via a terminal cardiac puncture, and the plasma was separated and stored at –80°C until further analysis. Three untreated mice were included with each group.

### Determination of antibiotic concentrations in plasma by liquid chromatography mass spectrometry (LC-MS).

The methodology for determining antibiotic concentrations by LC-MS is as described in Norville et al. (39). The plasma samples were mixed with an equal volume of internal standard solutions and 3 volumes of acetonitrile. After centrifugation, the supernatant was removed and reduced in volume to remove the acetonitrile using a centrifugal evaporator (Geneva Ltd., Ipswich, UK) and injected onto the LC-MS system consisting of an 1100 binary pump (Agilent Technologies UK Ltd., Wokingham, UK) and CTC PAL injector (Presearch Ltd., Basingstoke, UK) using an ACE-3-C18HL 20 by 2.1-mm column (Hichrom, Theale, UK) and a gradient mobile phase. The LC-MS system was controlled by Analyst Software (AB Sciex, UK). Calibration curves were developed for naive mouse plasma using reference standards of antibiotics co-trimoxazole (240 mg/kg Septrin; Aspen Pharma Trading Limited) and levofloxacin (5-mg/kg levofloxacin solution for infusion; Hospira UK Ltd.).

### Pharmacokinetic calculations.

The concentration-time data were analyzed using Phoenix WinNonlin (v8.0; Certara Inc.). Noncompartmental analysis was completed, with the sparse sampled option enabled. The elimination rate constant (*λ_z_*), terminal half-life (*t*_1/2_), clearance (CL), maximum concentration (*C*_max_), time of maximum concentration (*T_max_*), and area under the concentration-time curve (AUC) were calculated. The parameter estimates were subsequently used to calculate human equivalent doses used to treat Q fever, for the mouse studies.

### Infection of mice and antibiotic efficacy studies.

Mice were challenged with an aerosol produced from a 10-ml suspension of C. burnetii at a concentration of 1 × 10^7^ GE/ml using the AeroMP-Henderson apparatus. The aerosol challenge was generated using a six-jet Collison nebulizer (BGI, Walthan, MA) operating at 15 liter/minute containing PBS and an aerodynamic particle sizer (TSI Instruments, Ltd., Bucks, UK); this was monitored via the AeroMP management platform (Biaera Technologies, LLC, Frederick, MD). Reverse transcriptase PCR (RT-PCR) was performed on the aerosol challenge samples as described above. The presented dose was calculated using bacterial enumeration of the aerosol challenge, a derived respiratory minute volume of 19.9 ml estimated using the average weight of the animals (47), and the time the mice resided in the apparatus. Groups of 10 mice were treated 24 h pre- or postchallenge, continuing for 7 days postchallenge with oral doxycycline hyclate (105 mg/kg twice daily), intraperitoneal ciprofloxacin (22 mg/kg twice daily), intraperitoneal levofloxacin (40 mg/kg twice daily), and oral co-trimoxazole (48 mg/kg twice daily). Mice were observed for clinical signs (ruffled fur, arched back, dehydration, eyes shut, wasp-waisted appearance, immobility, and death) and weighed once daily for 14 days. Mice were culled on day 14 postchallenge, and the lungs and spleen were removed aseptically. Organs were weighed and homogenized using a Precellys24 homogenizer, and bacteria were enumerated by PCR. Sections of lung and splenic tissue were formalin fixed and embedded in paraffin wax. Sections were cut at 5 to 6 μm and stained with hematoxylin and eosin. Microscopic lesions were assessed and recorded for the presence of C. burnetii-associated lesions. Slides were assessed independently by two pathologists who were blind to the treatment.

### Statistical analysis.

All statistical analyses were performed using GraphPad Prism v7. Kaplan-Meier survival curves for the G. mellonella data were analyzed using a log-rank (Mantel-Cox) test. Mouse weight loss data were compared with controls using a two-way random matching ANOVA and Tukey’s multiple-comparison test. Organ weight was analyzed as a percentage of the total body weight. Organ bacterial burden (qPCR) data were transformed by log_10_ and then analyzed by a two-way random matching ANOVA. Statistical significance was indicated as follows: *, *P *< 0.05; **, *P *< 0.01; ***, *P*< 0.001.
